# Targeting the histone H3 lysine 79 methyltransferase DOT1L in MLL-rearranged leukemias

**DOI:** 10.1186/s13045-022-01251-1

**Published:** 2022-03-24

**Authors:** Yan Yi, Shenglei Ge

**Affiliations:** 1 Departments of Hematology, The Second Xiangya Hospital, Central South University, Changsha, 410011 Hunan People’s Republic of China; 2 Departments of Otolaryngology-Head and Neck Surgery, The Second Xiangya Hospital, Central South University, 139 Renmin Middle Street, Changsha, 410011 Hunan People’s Republic of China

**Keywords:** DOT1L, MLL-rearranged leukemia, Epigenetics, Histone modifications, Drug discovery, Small molecule inhibitors, Targeted protein degradation, Protein–protein interactions, Combined modality therapy

## Abstract

Disrupting the methylation of telomeric silencing 1-like (DOT1L)-mediated histone H3 lysine 79 has been implicated in MLL fusion-mediated leukemogenesis. Recently, DOT1L has become an attractive therapeutic target for MLL-rearranged leukemias. Rigorous studies have been performed, and much progress has been achieved. Moreover, one DOT1L inhibitor, EPZ-5676, has entered clinical trials, but its clinical activity is modest. Here, we review the recent advances and future trends of various therapeutic strategies against DOT1L for MLL-rearranged leukemias, including DOT1L enzymatic activity inhibitors, DOT1L degraders, protein–protein interaction (PPI) inhibitors, and combinatorial interventions. In addition, the limitations, challenges, and prospects of these therapeutic strategies are discussed. In summary, we present a general overview of DOT1L as a target in MLL-rearranged leukemias to provide valuable guidance for DOT1L-associated drug development in the future. Although a variety of DOT1L enzymatic inhibitors have been identified, most of them require further optimization. Recent advances in the development of small molecule degraders, including heterobifunctional degraders and molecular glues, provide valuable insights and references for DOT1L degraders. However, drug R&D strategies and platforms need to be developed and preclinical experiments need to be performed with the purpose of blocking DOT1L-associated PPIs. DOT1L epigenetic-based combination therapy is worth considering and exploring, but the therapy should be based on a thorough understanding of the regulatory mechanism of DOT1L epigenetic modifications.

## Introduction

Acute leukemias in which the mixed lineage leukemia (MLL, also called MLL1 or KMT2A) gene is translocated at 11q23 accounts for approximately 35–50% of pediatric acute myeloid leukemia (AML) and up to 80% of acute lymphocytic leukemia (ALL). In adults, MLL-rearranged leukemias account for approximately 5% of ALL cases and 5–10% of AML cases [1, 2]. Patients with MLL-rearranged leukemias generally have a worse response to therapy than those with non-MLL-rearranged leukemias. The prognosis of patients with MLL-rearranged leukemias is particularly poor. For example, for infants with MLL-rearranged ALL, the 5-year survival rate is only 34–39% depending on treatment protocols, whereas it is over 60% for those with wild-type MLL. The 5-year survival rate of patients with MLL-rearranged AML is less than 45%.

**Fig. 1:**
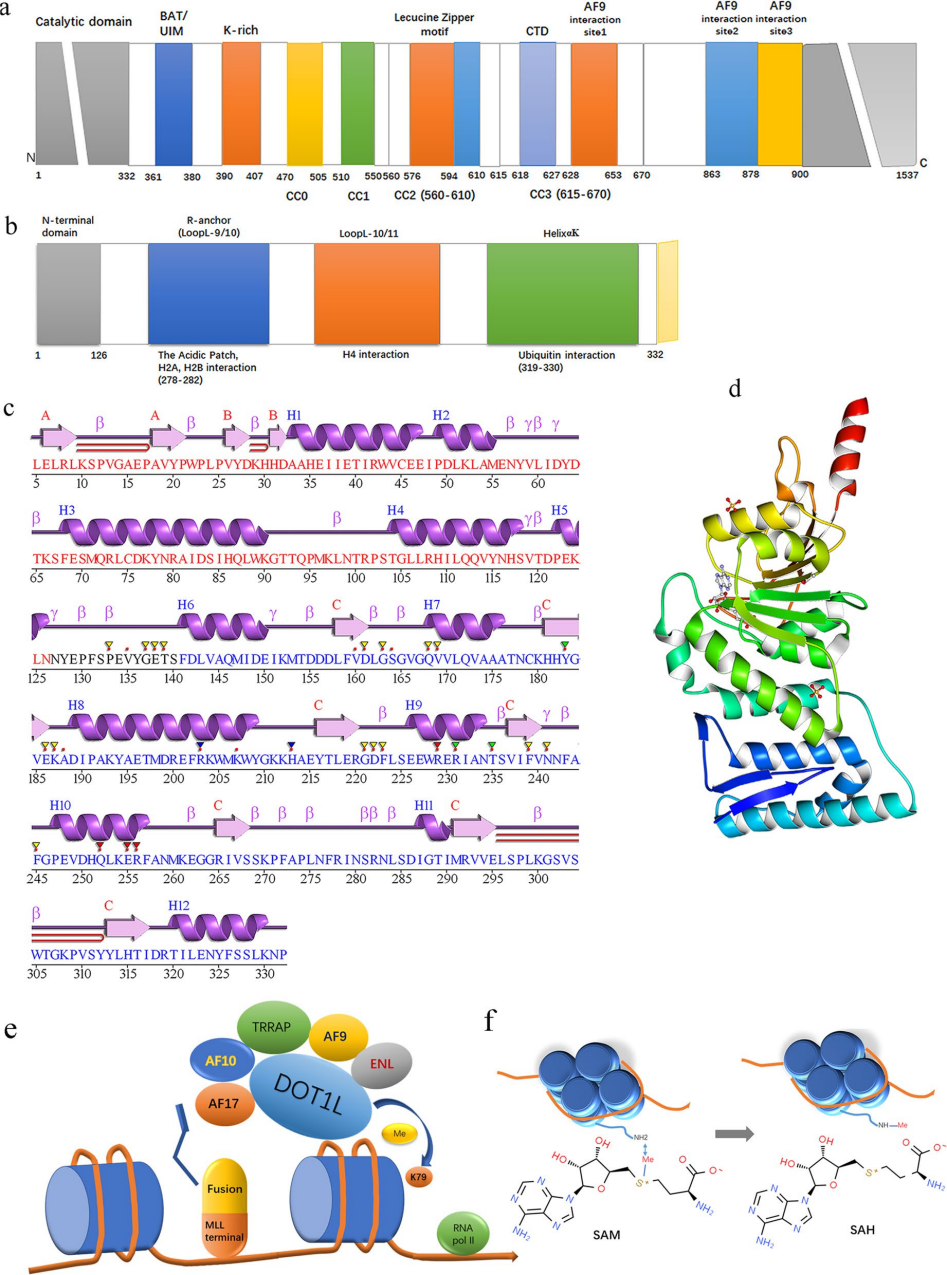
Schematic diagram of DOT1L. **a** Schematic diagram illustrating the domain structures of human DOT1L. DOT1L, which has more than 1530 amino acids, contains an N-terminal histone methyltransferase domain (gray) (residues 1–332) and a large C-terminal region of unknown functions. hDOT1L is predicted to contain 4 tandem coiled-coil (CC) domains (aa 419–670), and the CC1–CC3 domains are involved in AF10 binding. DOT1L has 3 separate AF9 binding sites as follows: site 1 (aa 628–653), site 2 (aa 863–877), and site 3 (aa 878–900). **b** Schematic diagram of the catalytic domain of human DOT1L. The sites of DOT1L interacting with the acidic patch, histone H2A, histone H2B, histone H4, and ubiquitin are presented. **c** Secondary structure plot for DOT1L 1nw3-PDBsum entry 1nw3-Chain A (328 residues). **d** Structure of the catalytic domain of human DOT1L (PDB: 1NW3). **e** Schematic diagram of DOT1L. DOT1L exists in a large macromolecular complex known as DotCom, which includes fusion partners such as ENL, AF9, AF17, and AF10, as well as the known Wnt pathway modifiers TRRAP, Skp1, and β-catenin. DOT1L is recruited to RNA Pol II through its associated protein network, which is not limited to the proteins of the DotCom complex, DOT1L-mediated H3K79 methylation and the subsequent activation of the target genes, resulting in leukemic transformation. **f** DOT1L mechanism of action. DOT1L transfers the S-methyl group of (S)-adenosyl-l-methionine (SAM) to the amino group of H3K79, producing a methylated substrate and (S)-adenosyl-l-homocysteine (SAH)

In MLL-rearranged leukemia, the N terminus of MLL fuses with the C terminus of various fusion partners, generating fusion proteins, which can bind to DNA or chromatin and induce leukemic transformation in hematopoietic stem and progenitor cells. Studies have shown that the disruptor of telomeric silencing 1-like (DOT1L) is involved in MLL fusion-driven leukemogenesis. DOT1L is the only histone methyltransferase that specifically targets nucleosomal histone H3 lysine 79 (H3K79) for mono-, di-, or trimethylation (H3K79me1, me2, or me3) [3]. Currently, the structure of the DOT1L protein is well understood (Fig. 1a–d). DOT1L is conserved from yeast to human. Human DOT1L has more than 1530 amino acids, containing an N-terminal histone methyltransferase domain (aa 1–332, a non-SET domain catalytic domain) and a long C-terminal region that may mediate interaction with fusion partners such as AF10, AF9, and eleven-nineteen lysine-rich leukemia (ENL). hDOT1L is predicted to contain 3 separate AF9 binding motifs and 4 tandem coiled-coil (CC) domains (aa 419–670), which are involved in AF10 binding. In addition, the contact of hDOT1L with the acidic patch, histone H2, H4, and ubiquitin has been demonstrated by structural analysis of its catalytic domain. DOT1L has been implicated in various biological processes, including gene transcription, heterochromatin formation, and DNA damage response. DOT1L is not only essential for mammalian embryonic development but is also postnatally needed, e.g., for cardiac function and hematopoiesis; otherwise, conditional deletion of the DOT1L ortholog in mice causes a variety of developmental deficiencies and is eventually lethal. DOT1L exists in a large macromolecular complex known as DotCom (DOT1L-containing complex), which includes several fusion partners, including AF10, ENL, AF9, and AF17, as well as the known Wnt pathway modifier TRRAP. Skp1, and β-catenin [4] (Fig. 1e). AF10 contributes to the conversion of H3K79me1 to di- and trimethylation, and AF9 and ENL can bind acetylated H3 to enhance the recruitment of DOT1L. The DOT1L-associated protein network is not limited to these proteins in the DotCom complex. DOT1L-mediated H3K79 methylation is closely associated with the biological function of various RNA polymerase II (RNA Pol II)-associated transcription elongation complexes, such as eleven-nineteen lysine-rich leukemia (ENL)-associated proteins (EAPs), AF4 family/ENL family/P-TEFb (AEP), and the super elongation complex (SEC) [5–7]. For example, MLL-ENL participates in the MLL-ENL/AEP and MLL-ENL/DOT1L complexes (ENL is one shared subunit of AEP and DotCom), which have mutually exclusive associations. Despite the complicated and indescribable relationships among these complexes, their interactions may eventually facilitate the transcriptional elongation of target genes that drive leukemogenesis. Interestingly, DOT1L has also been reported not only to promote transcriptional initiation by recruiting the initiation factor TFIID and enhancing H2B monoubiquitination but also to facilitate transcriptional elongation in a manner independent of H3K79 methylation [8, 9]. More importantly, DOT1L methyltransferase activity plays an important role in MLL fusion-mediated leukemogenesis, including MLL-AF9, MLL-AF10, MLL-AF4, and MLL-ENL fusions [10–12]. The interaction between MLL fusion partners and DOT1L has been demonstrated to promote transcriptional elongation and the aberrant recruitment of DOT1L to MLL target genes, such as the HOXA9 cluster and MEIS1, inducing the hypermethylation of H3K79 and constitutive gene expression at these ectopic loci that drive leukemogenesis. Recently, DOT1L has become a popular therapeutic target for MLL-rearranged leukemias. In this review, we describe the progress of DOT1L as a promising epigenetic therapy target for MLL-rearranged leukemias and discuss the existing concerns and prospects of the different therapeutic approaches to provide valuable guidance for DOT1L-associated drug development and future clinical treatments.

## DOT1L inhibitors

DOT1L transfers the S-methyl group of (S)-adenosyl-l-methionine (SAM) to the amino group of H3K79, producing a methylated substrate and (S)-adenosyl-l-homocysteine (SAH) [3] (Fig. 1f). Based on the catalytic mechanism, much effort has been dedicated to synthesizing small molecules that target the unique non-SET catalytic domain of DOT1L to inhibit DOT1L activity by displacing or blocking SAM from its binding site.

**Fig. 2:**
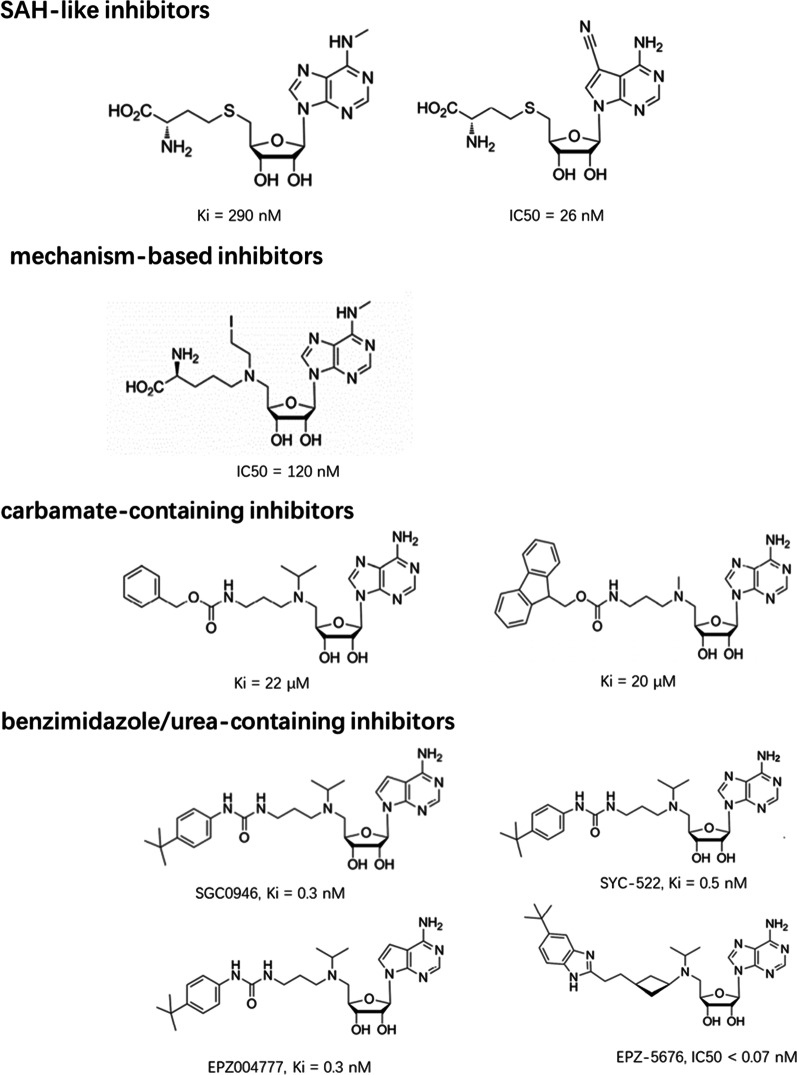
Classification and examples of the nucleoside DOT1L inhibitors

A common strategy is to develop SAM analogs by structurally modifying SAM; most nucleoside inhibitors have been synthesized according to this basis [13–17], and they are generally classified into four categories (Fig. 2). Among them, EPZ004777 and pinometostat (EPZ-5676) are the most thoroughly studied [18–21], but the pharmacokinetic properties of EPZ004777 are not conducive to its clinical development. Based on the structures of SAH and EPZ004777, EPZ-5676 was subsequently developed, which occupies the SAM-binding pocket of the DOT1L catalytic domain and induces conformational changes. EPZ-5676 has a lower Ki (0.08 nM), a higher selectivity (37,000-fold more selective for DOT1L over other histone methyltransferases), an increased potency (IC50 = 3.5 nM against MV4–11 cells), and an extended DOT1L-binding residence time compared to those of EPZ004777. EPZ-5676 treatment shows effective antitumor efficacy in a rat xenograft model with MLL-rearranged leukemia, while no significant weight loss was observed within 21 days of treatment [18–20]. Recently, direct cross-comparison among various inhibitors, including DOT1L, bromodomain and extraterminal domain (BET), dihydroorotate dehydrogenase (DHODH), P-TEFb, and menin-MLL1 inhibitors, has shown that they have functional diversity in blocking differentiation, and EPZ-5676 acts specifically on MLL-fused leukemia cell lines and significantly blocks differentiation [22]. With its superior potency, selectivity, and pharmacokinetics, EPZ-5676 has been in clinical trials since September 2012. Early clinical trials with EPZ-5676 in both pediatric and adult patients have been completed (NCT02141828 and NCT01684150) (Table 1). A total of 51 patients were enrolled and received continuous intravenous infusion of EPZ-5676 in 28-day cycles. The results showed that EPZ-5676 has modest clinical activity. The extent of H3K79me2 reduction and HOXA9 inhibition was variable and not necessarily correlated with clinical outcomes. EPZ-5676 was well tolerated, but continuous long-term administration could lead to drug resistance [19, 23]. This phase I clinical study also strongly suggests that EPZ-5676 in combination with other antileukemia agents is warranted.

**Table 1:** Current clinical trials of EPZ-5676

ClinicalTrials.gov identifier	Study title	Status
NCT01684150	A phase 1, open-label, dose-escalation and expanded cohort, continuous IV infusion, multi-center study of the safety, tolerability, PK and PD of EPZ-5676 in treatment relapsed/refractory patients with leukemias involving	Completed
NCT02141828	A phase 1 dose escalation and expanded cohort Study of EPZ-5676 in the treatment of pediatric patients with relapsed/Refractory leukemias bearing a rearrangement of the MLL gene	Completed
NCT03701295	Pinometostat and azacitidine in treating patients with relapsed, refractory, or newly diagnosed acute myeloid leukemia with 11q23 rearrangement	Completed Has Results
NCT03724084	Pinometostat with standard chemotherapy in treating patients with newly diagnosed acute myeloid leukemia and MLL gene rearrangement	Active, not recruiting

Furthermore, the common substructure of most nucleoside inhibitors is an adenosine or deazaadenosine, and the ribose fragments are rapidly degraded, resulting in poor pharmacokinetics. Therefore, developing DOT1L inhibitors with novel scaffolds as an alternative approach has attracted increasing attention, and most of the associated drug discovery approaches are described in this article (Table 2) [24–31]. The process of drug discovery may involve the application of multiple methods, and the integration of these methods will also benefit drug discovery. For example, virtual screening (VS) may be used as a complementary approach to reduce the initial amounts of compounds screened by high-throughput screening (HTS). Fortunately, multipronged approaches, such as the integration of fragment-based drug discovery (FBDD) and HTS, have been successfully applied to develop DOT1L inhibitors [31].

**Table 2:** Approaches to identify DOT1L inhibitors with novel scaffolds

Methods	Advantages	Examples
Virtual screening (VS)	Low cost and convenience	DC_L115 was identified by combining structure-based VS with biochemical analysis [24]
		As a target-specific scoring function, the SAM score was developed and combined with VS to successfully identify a novel class of DOT1L inhibitors with a scaffold of [1,2,4]-triazolo-[3,4-b][1,3,4]-thiadiazole [25]
		Two novel DOT1L inhibitors were identified by means of ligand-based and structure-based approaches [26]
		DOT1L inhibitors with a unique non-nucleoside scaffold were identified by structure-based VS adapted from a nucleoside-focused library [16]
Fragment-based drug discovery (FBDD)	The small, low complexity chemical fragments of 6–15 heavy atoms are screened to bind to or inhibit the activity of a target, moreover, the fragments are structurally understood	A weak fragment-based screening hit identified by SPR was cocrystallized with DOT1L and optimized using structure-based ligand optimization, resulting in a series of non-nucleoside inhibitors [27]
		In the context of a comprehensive DOT1L hit finding strategy, a non-nucleoside fragment mimicking key interactions of SAM bound to DOT1L was identified and followed by ligand optimization, resulting in a novel inhibitor [28]
High-throughput screening (HTS)	HTS platform routinely screens thousands of drug pairs in vitro setting	A luminescence-based coupled assay was used to measure DOT1L-catalyzed nucleosome methylation and miniaturized to perform HTS screening of the Novartis compound collection in 1536-well plates. Finally, a novel DOT1L inhibitor was identified [29]
		A HTS platform based on the AlphaLISA method was developed and screened a compounds library containing approximately 20,000 chemicals, and two promising candidate compounds were identified [30]
		A structure-guided optimization of a HTS hit led to the discovery of new potent DOT1L inhibitors for in vivo evaluations [31]

**Fig. 3:**
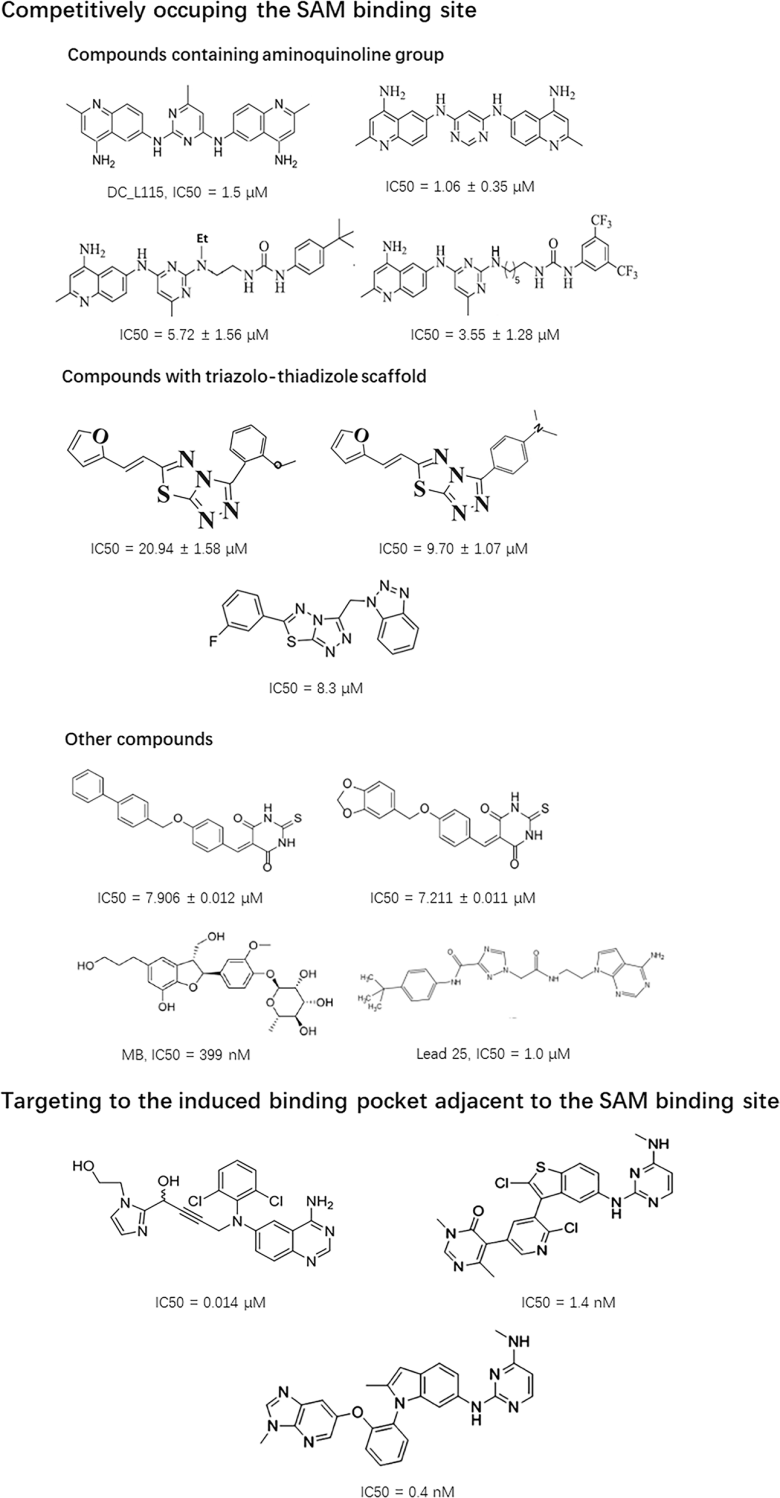
Classification and examples of non-nucleoside DOT1L inhibitors

We classified the non-nucleoside DOT1L inhibitors according to the SAM-competitive pattern (Fig. 3). Among these compounds, DC_L115, as the first novel non-nucleoside DOT1L inhibitor identified by structure-based VS, is predicted to efficiently bind to the SAM-binding site, but it has weak antiproliferation activity [24]. Based on DC_L115, a series of pyrimidylaminoquinoline derivatives 8(a–i) and 9(a–i) and bisaminoquinoline analogs 3(b–e) have been subsequently synthesized [32]. Due to their own limitations in either potency or selectivity (e.g., compounds 8 h and 9e exhibit better anti-DOT1L activity but poor selectivity against MLL-rearranged leukemia cells than that of 3a and 3e), these compounds need further optimization. Furthermore, a novel class of inhibitors with the same scaffold of [1, 2, 4]triazolo[3,4-b][1,3,4]thiadiazole (this scaffold aligns well with the furanoid sugar ring of SAM) substituted with different groups has been identified [25]. For example, triazolo and thiadiazole are substituted with a pyridyl or phenyl at positions 3 and 6, resulting in a series of analogs, several of which efficiently inhibit proliferation and induce the apoptosis of MV4-11 [30]. Recently, compounds 1MPS, 2MPS [26], massonianoside B (the first natural product that inhibits DOT1L) [33], and lead 25 [16] have also been identified to compete effectively with the SAM-binding site of DOT1L, indicating that their derivatives or analogs deserve further exploration as novel DOT1L inhibitors.

In addition, it is particularly worth mentioning that a novel class of non-nucleoside inhibitors developed by Novartis Institutes was reported to inhibit DOT1L by engaging in an induced binding pocket adjacent to the SAM-binding site and forming a conformation that is incompatible with efficient SAM binding; this occurs instead of the usual interactions within the SAM-binding pocket and is similar to the abovementioned non-nucleoside inhibitors [29]. Although several of these inhibitors have good pharmacokinetics, their potency and selectivity still need to be improved.

Above all, although a variety of DOT1L enzymatic inhibitors have been identified by scientists from Song Laboratory, Epizyme, Novartis Institutes, and Structural Genomics Consortium at University of Toronto, only a few have been evaluated by biochemical methods, cell-based assays, and in vivo studies. Of these, only EPZ-5676 has entered clinical trials, but its clinical effect is poor. Thus, alternative options to target DOT1L have increasingly attracted attention. The loss/degradation of DOT1L has also been proposed for MLL-rearranged leukemias [8].

## Loss/degradation of DOT1L

Studies have shown that DOT1L may not play a major role in transcriptional elongation in embryonic stem cells and erythroleukemic cells, and the loss of DOT1L may not affect the accessibly of global chromatin [8, 9]. Intriguingly, DOT1L, but not its catalytic activity, is necessary for neural differentiation, and DOT1L deletion, but not catalytic inactivation, further impairs transcriptional elongation upon SEC inhibition. Taken together, these results indicate that DOT1L has biological functions that are independent of H3K79 methylation, and degrading DOT1L instead of solely inhibiting its catalytic activities might be more important for cancer treatments. Therefore, it has been proposed that the loss/degradation of DOT1L could be beneficial for cancer therapeutics [8, 9].

In recent years, targeted protein degradation (TPD) has attracted extensive attention. With the potential to tackle therapeutically modulate proteins that have been difficult to target with traditional small molecules, antisense oligonucleotides, or genetic perturbations such as RNA interference and CRISPR/Cas9 genome editing, TPD may open a new avenue for the target-specific control of DOT1L in regard to its function or abundance.

### Proteolysis-targeting chimeras (PROTACs)

**Fig. 4:**
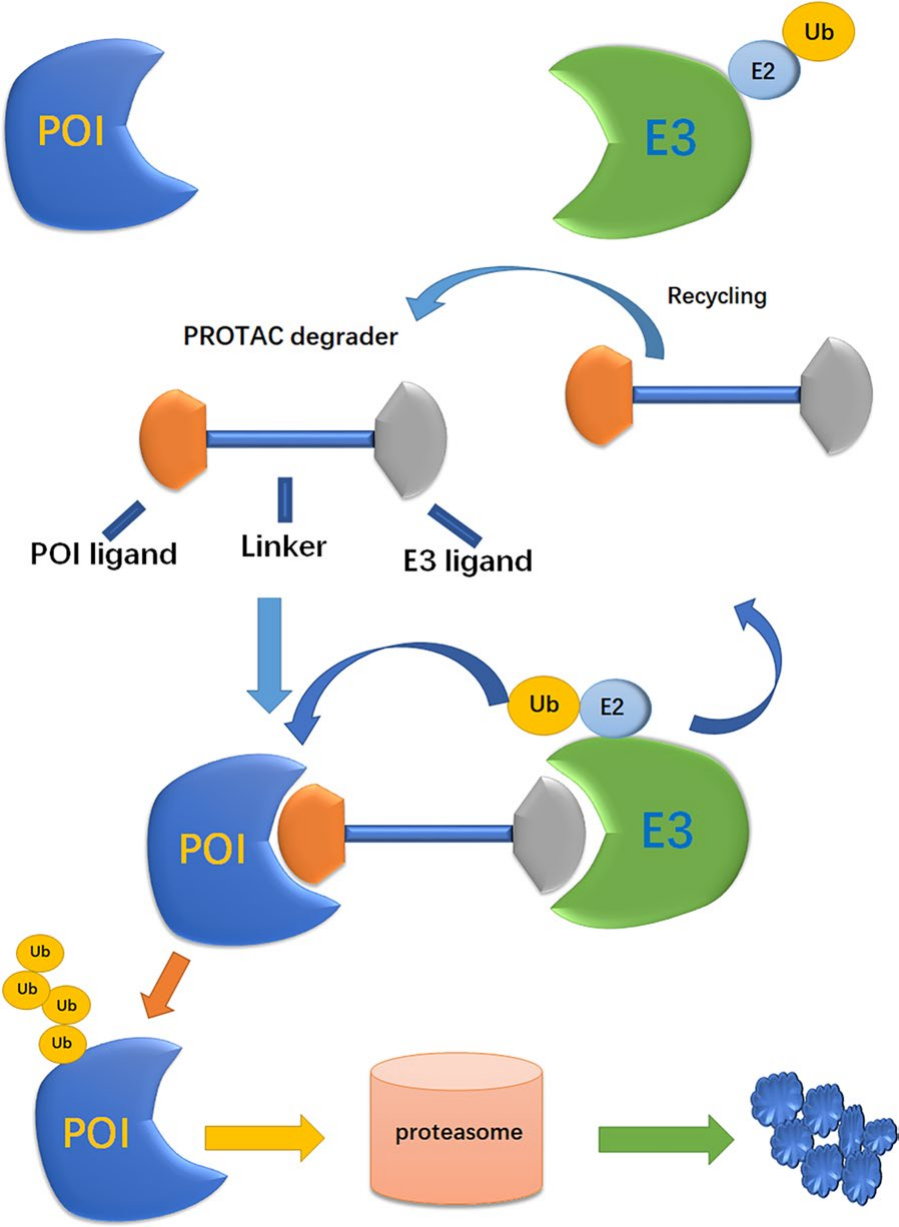
Schematic representation of protesolysis-targeting chimera (PROTAC) degraders. A protein of interest (POI) can be degraded by the ubiquitin–proteasome system, which is mediated by a PROTAC and consists of a POI ligand connected to an E3 ligand via a linker. The PROTAC molecule is recycled to induce the next round of degradation

A key focus of TPD is the development of heterobifunctional small molecule degraders such as proteolysis-targeting chimeras (PROTACs), which contain two linked moieties: One moiety binds to a protein of interest (POI) and another binds to an E3 ubiquitin (E3) ligase. PROTACs recruit the E3 ligase to the POI and cause proximity-induced ubiquitination and degradation of the POI by the ubiquitin proteasome system, after which the PROTACs are recycled to target another copy of the POI (Fig. 4). PROTACs have many advantages over traditional small molecule inhibitors, including the potential to target undruggable proteins, the capacity to overcome drug resistance, and a high selectivity by using tissue- and/or tumor-specific E3 ligase ligands [34, 35]. Over the past 5 years, a large variety of heterobifunctional small molecule degraders have been developed for solid tumors and hematological malignancies. Remarkably, the first two PROTACs, which are named ARV-110 and ARV-471, respectively, entered clinical testing in 2019 ( NCT03888612 and NCT04072952).

### Other approaches

#### Molecular glues

Molecular glues can bind both an E3 ligase and a neosubstrate and enhance their interactions, resulting in the degradation of the POI. With low molecular weight, molecular glues are more likely to produce favorable pharmacokinetic properties, such as oral bioavailability.

#### Hydrophobic tag-mediated degradation

Hydrophobic tag-mediated degradation starts in the endoplasmic reticulum and differs from PROTAC-mediated degradation. Hydrophobic tagging links a POI ligand with a highly lipophilic moiety that activates the unfolded protein response, thereby inducing POI degradation. Many hydrophobic tags have been identified recently. For example, the dTAG system, which combines with a PROTAC derived by linking an FKBP12-F36V binder to a CRBN ligand, was created and exhibits a superior antiproliferative effect of pan-BET bromodomain degradation over selective BRD4 degradation [36], and it can also lead to the acute degradation of ENL [37].

### Designing DOT1L degraders

However, thus far, there is no report about PROTACs against DOT1L. In general, E3 ligands, POI ligands, linker attachments, and linker types and lengths are very important for the formation of sufficiently stable POI-PROTAC-E3 ternary complexes. Likewise, these problems need to be solved one by one to design an effective PROTAC to degrade DOT1L. Perhaps we can effectively develop it via some advanced TPD technical tools, such as bioPROTACs, the dTAG system and antibody–PROTAC conjugates [38]. Overall, this task requires us to thoroughly understand the structure and spatial conformation of DOT1L, develop specific/selective E3 ligands, and select the optimal linkers and proper degradation reaction conditions. Here, we also emphasize that the molecular mechanism of DOT1L should be further elaborated, especially whether its cross talk with H2Bub may affect the formation and stability of the ternary complex to degrade DOT1L. In addition, other factors, such as the half-life of the PROTACs, the abundance of the selected E3 ligase in cells, and the toxicity and cell permeability of degraders, also deserve attention.

To date, many ternary complexes, such as the POI-PROTAC-VHL, POI-PROTAC-CRBN, and POI-PROTAC-BIRC2 ternary complexes, have been developed. Their crystal structures, including the protein–protein interactions, have been analyzed, which should contribute to generating more effective PROTACs for clinical treatments. Recent advancements in small molecule degraders encouraged us to explore the design of an effective PROTAC or molecular glue to degrade DOT1L as an alternative approach to enzymatic inhibitors in MLL-rearranged leukemias. Although the results, such as the failure to assemble a stable ternary complex, may not be as satisfactory as expected, there may be an encouraging breakthrough similar to BRD4-targeting PROTAC, which can effectively degrade BRD4 [39, 40], so the topic is worth exploring, and we are looking forward to the gratifying research results in the field of PROTACs against DOT1L in MLL-rearranged leukemias in the near future.

## Disruption of protein–protein interactions (PPIs) involved in DOT1L epigenetic regulation (Fig. 5)

**Fig. 5:**
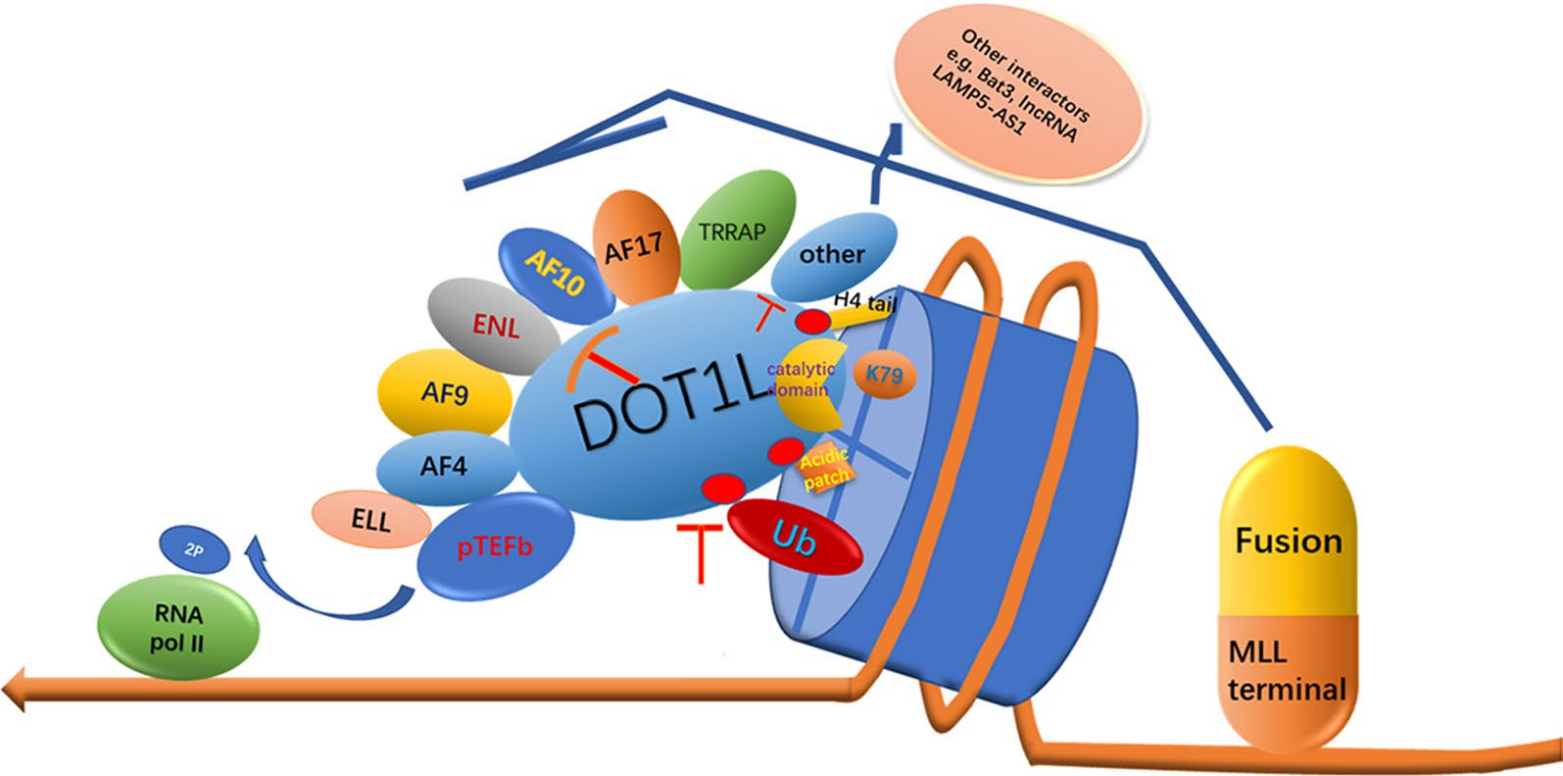
Overview of disrupting PPIs that involve the epigenetic regulation of DOT1L. Disruptions of PPIs include DOT1L interaction with fusion partners, ubiquitin, histone cross talk, the acidic patch, and other interactors, such as Bat3 and lncRNA LAMP5-AS1

Recently, more reports have focused on PPIs involving DOT1L epigenetic regulation because this targeted disruption not only ensures that the methylation activity of DOT1L is not affected but also has higher selectivity for leukemia cells than inhibition of DOT1L enzymatic activity.

### Disrupting the association of DOT1L with fusion partners

#### Disrupting the DOT1L-AF9/ENL interaction

**Fig. 6:**
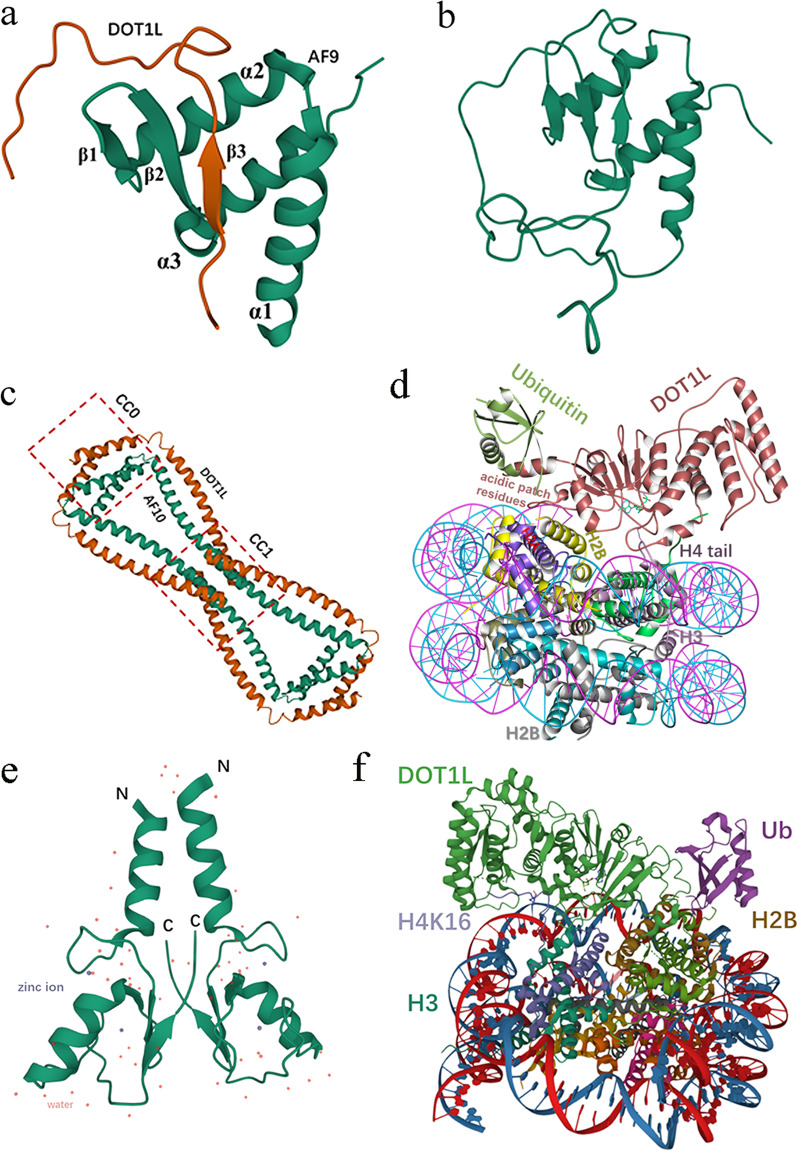
Close-up view of DOT1L in complex with other epigenetic regulators. **a** Solution NMR structure of DOT1L in complex with AF9 (DOT1L-AF9) (PDB: 2MV7). **b** Solution structure of the AF4–AF9 complex (PDB: 2LM0). **c** Crystal structure of the coiled-coil domains of human DOT1L in complex with AF10 (PDB: 6JN2). **d** Structure of the DOT1L-H2BK120ub nucleosome complex (PDB: 6NN6). **e** Crystal structure of the RNF20 RING domain (PDB: 5TRB). **f** Active DOT1L bound to the H4K16ac nucleosome (PDB: 7K6Q)

AF9 and ENL, which are subunits, coordinate SEC and DotCom stabilization and activity in abnormal regions of the genome [41]. AF9 and ENL recruit DOT1L (and possibly SEC) to target genes via the recognition of H3K9ac (Histone H3 acetyl Lys9) by their YEATS (Yaf9, ENL, AF9, Taf14, and Sas5) domain, and the subsequent deposition of H3K79 methylation promotes active transcription in MLL leukemogenesis [42]. The interactive sites between AF9 and DOT1L have been roughly mapped [43, 44]. DOT1L has three separate motifs for binding with AF9 as follows: site 1 (aa 628–653), site 2 (aa 863–878), and site 3 (aa 877–900) (Fig. 1a). The functional domain of AF9 (aa 499–568) has also been demonstrated to produce the optimal nuclear magnetic resonance (NMR) spectra with all three DOT1L motifs and to form a DOT1L-AF9 complex with a mixed α-β structure, similar to the AF4-AF9 complex (PDB: 2LM0) (Fig. 6a, b) [45, 46]. The point mutation of AF9 and the DOT1L-binding domain may weaken their interaction and reduce DOT1L recruitment, thus affecting the hematopoietic transformation of MLL-AF9. Therefore, disrupting the DOT1L-AF9/ENL interaction may be a promising therapeutic strategy with fewer potential side effects than that of the enzymatic inhibition of DOT1L [2, 47, 48]. The conserved binding motifs may be used as scaffolds to design small potent compounds. For example, a DOT1L 10-mer peptide has been found to inhibit AF9- and ENL-DOT1L interactions (IC_50_ values of 0.49 μM and 1.34 μM, respectively) as well as MLL1-AF9 transformations [47, 48]. Based on the DOT1L 7-mer peptide (aa 865–871), a series of peptidomimetics has been synthesized to effectively protect against the interaction between AF9/ENL and DOT1L with IC_50_ values as low as 57 nM [47]. Interestingly, because AF9 and ENL exist in two mutually exclusive complexes, namely AF9/ENL-DOT1L and AF9/ENL-AF4-pTEFb [43, 46], small molecules, such as the DOT1L 10-mer peptide and AF4 14-mer peptide, can block AF9/ENL interactions with both AF4 and DOT1L by binding the conserved hydrophobic domain of AF9/ENL, thus affecting the function of these complexes and MLL fusion-mediated leukemogenesis [2, 44].

#### Disrupting DOT1L-AF10 interactions

AF10 has been shown to interact with DOT1L, resulting in a significant enhancement of H3K79me2/3 markers compared to that of DOT1L alone [49]. Structural and functional analyses have shown that there is a conserved binding mode between the coiled-coil domains of DOT1L and the octapeptide motif-leucine-zipper (OM-LZ) region of AF10 (AF10^OM−LZ^), which has been confirmed to be essential for both MLL-AF10- and clathrin assembly lymphoid myeloid leukemia (CALM)-AF10-induced leukemogenesis (Fig. 6c) [11, 50, 51]. hDOT1L is predicted to contain 4 tandem coiled-coil (CC) domains, consisting of CC0 to CC3 (aa 419–670) (Fig. 1a). The CC1-CC3 domains are involved in AF10 binding, and CC1 may have the highest affinity toward AF10^OM−LZ^. Although AF9 and AF17 (AF17 also has a conserved C-terminal OM-LZ domain and may have a similar interaction as AF9) compete with AF10 to interact with DOT1L, all of them play the oligomerization role that is proposed for MLL [52, 53]. The DOT1L-AF10 binding interface may be an attractive therapeutic target, similar to the disruption of the DOT1L-AF9 interaction. Moreover, successful disruption of glioma-amplified sequence 41 (GAS41)-AF10^OM−LZ^ binding by the inhibitory peptides results in a lower HOXA expression [54], which provides a template reference for inhibiting the DOT1L-AF10 interaction. Thus, the development of drugs that target DOT1L-AF10^OM−LZ^ is worth exploring.

### Blocking histone cross talk between H3K79me by DOT1L, H2B ubiquitination, and H4 acetylation on nucleosomes

It has been shown that histone H3 methylation, H2B ubiquitination, and H4 acetylation on nucleosomes work together to ensure that the chromatin structure is properly regulated. Cross talk between these modifications and enzymes that deposit them are crucial for gene transcription. Understanding the detailed mechanisms of their cross talk, including DOT1L regulation through histone acetylation and ubiquitination, would contribute to targeting DOT1L in MLL-rearranged leukemias.

#### Blocking PPIs within the DOT1L-H2BK120ub nucleosomes

DOT1L interacts with histone H4, the acidic patch, DNA, and ubiquitin to form the DOT1L- Histone H2B lysine 120 monoubiquitination (H2BK120ub) nucleosome complex [54–57] (Fig. 6d). The interactions are present regardless of the H2BK120 ubiquitination status. Cross talk between H2B ubiquitination and H3 methylation that is induced by DOT1L is a popular research topic. H2BK120ub has been shown to regulate DOT1L from a poised state to an active state to access H3K79 for methylation or from a flexible to stabilized status for H3K79 hypermethylation via a conformational rearrangement that involves multiple interactors, such as the acidic patch and the H4 tail [55, 58]. Point mutations, such as DOT1L aK helix mutants (F326A and L322D) and DOT1L R278E/R282E, have been shown to abrogate the stimulation of DOT1L by H2BK120ub, resulting in significantly reduced H3K79 methylation [58]. The DOT1L conserved binding motifs may be regarded as a promising lead structure for designing small molecules that disturb the interactions of DOT1L with ubiquitin and acidic patches. However, H2BK120ub and the acidic patch may mediate similar interactions with many chromatin proteins other than DOT1L. Therefore, the specificity and selectivity of these small molecules require more attention to prevent side effects or off-target effects.

#### Targeting histone H2B ubiquitin ligase RNF20

In particular, histone H2B E3 ubiquitin ligase ring finger protein 20 (RNF20)-mediated H2B ubiquitination is reported to be enriched in the body of MLL fusion target genes such as HOXA9 and MEIS1, correlating with sites of H3K79 methylation by DOT1L and transcription elongation [59]. The suppression of RNF20 in diverse models of MLL-rearranged leukemias resulted in reduced H3K79 methylation by DOT1L and the inhibition of cell proliferation, suggesting that reducing RNF20-mediated H2B ubiquitination induces an antileukemic effect. In addition, targeting RNF20 is reported to be clinically useful for overcoming acquired resistance to DOT1L inhibitors [59]. We speculated that p53, which was demonstrated to be closely related to drug resistance, might be involved in this interesting finding. RNF20 and/or the RNF20/RNF40 complex have been identified as transcriptional coactivators at the promoters of p53 target genes [60]. The suppression of RNF20-mediated H2B ubiquitination leads to reduced transcription of these genes, thus affecting the function of p53. Of course, the above hypothesis needs to be confirmed.

In summary, RNF20 is a possible therapeutic target in MLL-rearranged leukemias, but this method faces many challenges; for example, the therapeutic effect of RNF20 inhibition is sometimes not obvious [60]. Notably, although RNF20 is an E3 ubiquitin ligase, it has not been applied to assemble the POI–PROTAC–E3 ternary complex like other E3 ligases, such as VHL, RNF4, and RNF114, which have been reported to participate in PROTACs. Therefore, understanding RNF20 cross talk with DOT1L and H2B ubiquitination on nucleosomes may contribute to developing the optimal E3 ligase for the abovementioned DOT1L degraders.

#### Blocking the cross talk between DOT1L and H4K16 acetylation

Furthermore, the activity of DOT1L has been shown to be associated with histone acetylation. Acetylation of H4 lysine 16 (H4K16ac), which is perhaps the most critical modification on H4, may play a crucial role in H3K79 di- and trimethylation. Cross talk between H4K16ac and H3K79me by DOT1L has been described by a combination of biochemical, structural, and functional approaches [61]. H4K16 acetylation has been shown to allosterically stimulate yeast DOT1L in a manner that is distinct from H2BUb but also coordinates with H2BUb. H4K16ac directly stimulates the catalytic activity of DOT1L on nucleosomes, obstructs the contact of the H4 tail and the acidic patch, and further prevents the SIR complex from binding to chromatin. In addition, H4K16ac plays a role in restricting the conformation of DOT1L and orients the active site over H3K79 for catalysis. The cryo-EM structure of DOT1L-H4K16ac and the DOT1L-unacetylated H4 complex revealed that acetylation of H4K16 and its interaction with AcG loop residue I261 of DOT1L favors the stabilization of the DOT1L-H4 tail interface (Fig. 6f). DOT1L mutants such as H347E-H355E and H355E, which play essential roles in histone H4 binding, lead to an almost complete loss of methylation activity on unmodified and H4K16ac nucleosomes. The DOT1L I261E mutant resulted in reduced stimulation by H4K16ac, whereas stimulation by H2BUb was not affected [61]. These binding sites may be regarded as a promising lead structure for designing small molecules to selectively and specifically disturb the cross-talk between DOT1L and the methylation of H3K79, H4K16ac, and H2Bub. Furthermore, developing small compounds that target both the H4 tail-binding region and the active site tunnel may be more effective, similar to the way that many DOT1L enzymatic inhibitors restructure the F131 loop [24–29]; this causes histone H3 to have a conformational instability, eventually blocking the SAM-binding site and the lysine-binding tunnel.

Importantly, many other proteins have been reported to interact with DOT1L (Fig. 5). For example, DOT1L has been reported to directly bind to the phosphorylated CTD of the actively transcribing RNA Pol II through a conserved region of DOT1L (aa 618–627), which is termed the CTD-binding patch [62] (Fig. 1a). This interaction helps DOT1L recruit RNA Pol II-targeted regions, thus mediating H3K79 methylation. Bat3 has been shown to colocalize and interact with both DOT1L and H3 to increase DOT1L activity [63]. The lncRNA LAMP5-AS1 has been reported to facilitate DOT1L activity and upregulate H3K79me2/me3 by directly binding the Lys-rich region of the DOT1L catalytic domain [64]. Blocking DOT1L interactions with these proteins may also contribute to inhibiting H3K79 methylation and enhancing therapeutic efficacy.

However, to date, investigations on blocking PPIs to understand their molecular mechanism are limited to in vitro biological investigations, but the availability of X-ray or NMR structures and biochemical assays would further facilitate medicinal chemistry studies of these PPI inhibitors in the treatment of MLL-rearranged leukemias.

## Cooperation between DOT1L inhibition and other therapeutic regimens in MLL-rearranged leukemia treatments

**Fig. 7:**
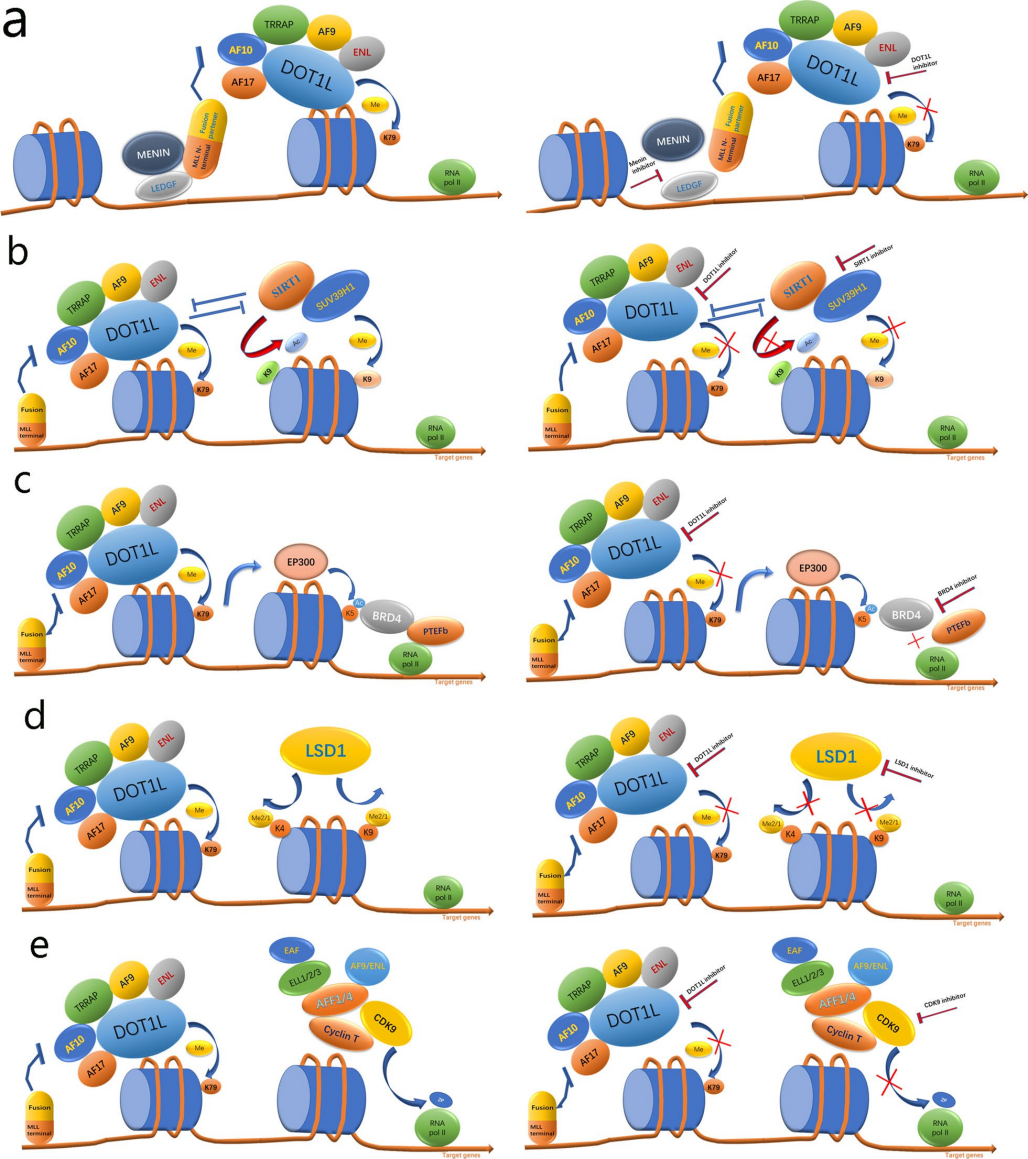
Schematic model for DOT1L and other epigenetic regulator-mediated gene transcription mechanisms and pharmacological inhibition. **a** Schematic model of the possible DOT1L and menin inhibition-mediated downregulation of MLL fusion target gene expression in MLL-rearranged leukemias. The intact MLL complex stably binds to its target genes via menin and recruits the DOT1L complex to drive target gene expression. Dual inhibition may disrupt the integrity of the oncogenic MLL complex and target gene activation. **b** Working model of possible DOT1L inhibition and SIRT1 activation. SUV39H1 and SIRT1 increase H3K9 methylation and decrease H3K9 acetylation, and DOT1L inhibits the chromatic localization of SIRT1 and SUV39H1, thereby maintaining an open chromatin state with elevated H3K9 acetylation, elevated H3K79 methylation, and minimal H3K9 methylation at MLL fusion protein target gene promoters. **c** Schematic model of the dual inhibition of DOT1L and BRD4. BRD4 binds K3K27ac and associates with SEC to allow phosphorylation and activation of RNA Pol II. DOT1L methylates H3K79 via H4 acetylation mediated by EP300, which regulates BRD4 binding to chromatin and the subsequent transcriptional gene expression in MLL leukemogenesis. **d** Schematic model for the dual inhibition of DOT1L and LSD1. LSD1 demethylates mono- and dimethylated lysine residues 4 and 9 on histone H3 (H3K4me1/2 and H3K9me1/2, respectively). LSD1 has been implicated as a drug target for leukemia, and dual inhibition of DOT1L and LSD1 has a synergistic effect. **e** Schematic model of dual inhibition of DOT1L and SEC or CDK9. As the largest pTEFb complex, the SEC complex is composed of CDK9, cyclin T, ELL1/ELL2, AFF1/AFF4, and ENL/AF9. Several translocation partners of MLL associate with ELL and pTEFb in SEC and lead to the misregulation of transcriptional elongation, such as HOX genes. Dual inhibition would improve efficacy by targeting these two parts of a developing and complex chromatin signaling pathway, including transcriptional elongation and histone methylation

Combinatorial intervention or a multipronged targeting approach, such as the integration of multiple modified histone signals, has attracted increasing attention in leukemia treatments. The results of early clinical trials of EPZ-5676 have shown that the combination of EPZ-5676 and other antileukemia agents is necessary, and many studies have suggested that DOT1L inhibitors in combination with other regimens may offer better therapeutic effects for MLL-rearranged leukemias than that of non-combinational treatments (Fig. 7).

### Combination of DOT1L inhibitor with other novel epigenetic therapies

#### *DOT1L and MLL scaffold protein: menin (Fig. **7**a)*

Menin binds to MLL through five amino acid sequences near the N terminus of MLL, and the MLL-menin interaction is crucial for MLL fusion-driven gene expression. Blocking this interaction has been demonstrated to be a potential therapeutic strategy for MLL-rearranged leukemias. To date, many inhibitors have been identified, such as MI-2-2 [65], VTP50469 [66], and SNDX-5613 (ClinicalTrials.gov number NCT04065399). The combination of MI-2-2 and EPZ-5676 or EPZ004777 markedly enhances the treatment efficacy in MLL-rearranged leukemias compared to either single agent and does not produce a significant effect on normal hematopoiesis or leukemias without MLL rearrangements. Moreover, the DOT1L-menin inhibitor combination has been shown to be equally or more effective than the recently published combination of a DOT1L inhibitor and cytarabine [67]. Therefore, combined therapy with DOT1L and menin inhibition, as recently used in nucleophosmin 1 (NPM1)-mutant leukemia [68], may be a promising therapeutic strategy in MLL-rearranged leukemias.

#### DOT1L and SIRT1 (Fig. 7b)

As a member of the sirtuin family, silencing information regulator 2 related enzyme 1 (sirtuin1, SIRT1) is an NAD-dependent protein deacetylase that links transcriptional regulation. DOT1L inhibits the localization of a repressive complex composed of SIRT1 and the H3K9 methyltransferase suppressor of variegation 3–9 homolog 1 (SUV39H1) in chromatin, thereby maintaining an open chromatin state with elevated H3K9 acetylation and minimal H3K9 methylation at the MLL fusion target genes [69, 70]. Conversely, inhibiting DOT1L in MLL fusion leukemias, such as MLL-AF9 leukemia, leads to a SIRT1-dependent decrease in H3K9 acetylation, gain of H3K9 methylation, and loss of chromatin accessibility at MLL fusion target genes [71]. The necessity of SIRT1 for establishing a chromatin-repressive state upon DOT1L inhibition suggests that SIRT1 activation might enhance the efficacy of a DOT1L inhibitor. SRT1720, a small molecule activator of SIRT1, has been demonstrated to sensitize MLL-rearranged leukemias, such as MLL-AF9 and MLL-AF4, to EPZ4777 and enhance its antiproliferative activity [71]. In addition, EPZ4777 and SRT1720 together have no synthetic toxicity in non-MLL-rearranged leukemia cells. Therefore, patients with MLL-rearranged leukemias may benefit from the combination of DOT1L inhibitors and SIRT1 activators.

#### DOT1L and BRD4 (Fig. 7c)

As a member of the BET family, bromodomain containing 4 (BRD4) reads acetyl groups on histone lysines. BRD4 and DOT1L mainly exist in separate protein complexes, and there is a high level of cooperation and interdependency between them. For example, DOT1L methylates histone H3K79 via H4 acetylation, which is mediated by E1A binding protein (EP300) and regulates the binding of BRD4 to chromatin and the subsequent transcriptional gene expression in MLL leukemogenesis. Dual targeting of DOT1L and BRD4 by SGC0946 and I-BET results in a greater synergistic efficacy in MLL-FP leukemia than that of single targeting [72]. In addition, BET inhibitors, as a promising epigenetic therapeutic avenue, inhibit the recruitment of BET to chromatin and the subsequent transcription of key genes (BCL2, C-MYC, and CDK6) through the displacement of BRD3/4, PAFc, and SEC components from chromatin [73]. Wnt signaling has been shown to promote primary and acquired BET resistance in leukemia [74], but it is not clear whether DOT1L inhibition alleviates the resistance of BET with the dual inhibition of DOT1L and BRD4.

#### DOT1L and LSD1 (Fig. 7d)

The lysine-specific histone demethylase 1 (LSD1) eraser targets H3K4. Overexpression of LSD1 reduces H3K4me1 and H3K4me2, which are key activating marks in normal cell growth and differentiation, resulting in gene repression [75]. LSD1 has been implicated as a drug target for leukemia, and multiple LSD1 inhibitors are in clinical development [76, 77], such as Iadademstat [78], INCB059872 (NCT04061421), and CC-90011 (NCT03850067). Remarkably, the LSD1 inhibitor ORY-1001 works synergistically with DOT1L inhibitors, such as EPZ-5676, SYC-522 or SGC0946, against MLL-rearranged leukemias [78, 79]. This synergism is presumably attributed to the imbalance of H3K79 hypermethylation and H3K4 methylation.

#### DOT1L and DNA methyltransferase (DNMT)

Epigenetic dysregulation in MLL-rearranged leukemias has been demonstrated to be associated with genome-wide hypomethylation, increases in methylation entropy, and loss of methylation sensitivity [80]. The murine hematopoietic stem cell DNA methylome has revealed large undermethylated canyons, and many canyon genes are regulated by histone modifications, such as the methylation of H3K27, H3K4, and H3K79 [81]. H3K79me2 is highly enriched in methylation canyons, and the most prominent canyons are those that expand with DNMT3a loss. Moreover, canyons coated with H3K79me2 are highly enriched for HOX cluster gene expression. The functional interaction between H3K79me2 and altered DNA methylation has been at least partly explained in DNMT3A*-*mutant acute myeloid leukemia (AML) [82]. Mutations in DNA methylation (e.g., DNMT3A) and demethylation (e.g., TET2) are frequent in patients with hematologic malignancies. DNMT inhibitors, such as 5-azacytidine and decitabine, have been used in AML and myelodysplastic syndrome (MDS). Remarkably, the synergistic antiproliferative activity of EPZ-5676 combined with azacitidine or decitabine has been confirmed, and this synergy is also specific to MLL-rearranged leukemia [83]. Recently, phase Ib/II trial studies of EPZ-5676 and azacytidine, as therapeutic agents, in MLL-rearranged leukemias were completed (ClinicalTrials.gov Identifier: NCT03701295) (Table 1).

#### DOT1L and SEC

P-TEFb phosphorylates the CTD of RNA Pol II on Ser5 and/or Ser2 to facilitate transcriptional elongation in MLL leukemogenesis [84], and this phosphorylated CTD is also necessary for the DOT1L interaction with RNA Pol II [62]. As the largest P-TEFb complex, the SEC complex is mainly composed of cyclin-dependent kinase 9 (CDK9), cyclin T1, ELL1/ELL2, AF4/FMR2 family member 1/4 (AFF1/AFF4), and ENL/AF9 (Fig. 7e). As one core component, active CDK9 assembles into multiple elongation complexes, such as the SEC and BRD4-containing elongation complex, and CDK9 is critical for processes in RNA Pol II transcription, including initiation, elongation, and termination. Inhibiting CDK9 activity has been considered a promising strategy for AML, and several CDK9 inhibitors have entered clinical trials. Several translocation partners of MLL associate with ELL and P-TEFb in SEC and lead to the misregulation of transcriptional elongation, such as the overexpression of HOX genes [85, 86]. Because transcriptional elongation and histone methylation are two parts of a complex and developmental chromatin signaling pathway, targeting both avenues would improve the efficacy of treating MLL-rearranged leukemias [85]. For example, SEC inhibition, such as KL-2, combined with DOT1L loss has been shown to further slow RNA Pol II processivity in embryonic stem cells [8]. Furthermore, DOT1L has been shown to inhibit SEC-dependent transcription by competing with AFF4 for binding to ENL/AF9 (AF9 and ENL are common subunits of both SEC and DotCom), thus affecting HIV-1 transcription [7]. Therefore, DOT1L inhibition combined with the inhibition of SEC or CDK9 may increase the therapeutic effect of a single DOT1L inhibition in MLL-rearranged leukemias. However, the synergistic effect must still be experimentally confirmed.

#### DOT1L and AF4 fusion protein

Given the collaborative effects of AEP and DOT1L on MLL fusion-dependent gene activation, simultaneous inhibition of the MLL fusion-AF4 complex and DOT1L may have a greater effect on MLL-rearranged leukemias than that of inhibiting each component individually [6]. Because menin is necessary for AF4 recruitment, an MLL-menin interaction inhibitor would induce the dissociation of the MLL fusion-menin-AF4 complex from target chromatin and affect gene activation by AF4 [87], thereby explaining the complementary activities of DOT1L and menin inhibitors in MLL-rearranged leukemia.

#### DOT1L and ENL fusion protein

ENL binds to acetylated histone H3 via its YEATS domain and colocalizes with H3K9ac and H3K27ac on the promoters of actively transcribed genes that are essential for ENL-dependent leukemia [37, 88]. The ENL YEATS domain also helps to stabilize the association of SEC and DotCom with DNA. XL-13 m, an ENL YEATS-selective inhibitor, engages with endogenous ENL and blocks recognition of acetylated H3. XL-13 m cooperates with the downregulation of oncogenes in MLL-rearranged leukemia that is induced by BET or DOT1L inhibition [48], suggesting that these interventions are synergistically cooperative.

### DOT1L and standard chemotherapy agents

EPZ-5676 has been reported to act synergistically with the AML standard of care agents Ara-C and daunorubicin, and this combinational therapy not only enhances antiproliferative activity over single agents in MLL-rearranged leukemia cells, such as MOLM-13 and MV4-11 cells, but also enhances the ability to induce cell apoptosis and differentiation without similar effects on non-rearranged leukemia cells, such as SKM-1 [83]. Treatment with EPZ-5676 in combination with standard chemotherapy drugs, such as daunorubicin hydrochloride and cytarabine, for patients with newly diagnosed AML and MLL-rearranged leukemias is currently being evaluated (ClinicalTrials.gov Identifier: NCT03724084) (Table 1).

### DOT1L and targeted therapy

#### DOT1L and MYC

MYC, an upregulated oncogenic transcription factor in MLL-rearranged leukemias, represents an important therapeutic target. C-Myc mediates the suppression of different tumor suppressor miRNAs in MLL-rearranged leukemias, such as miR-150 and miR-26a [88–90], and MYC restoration may participate in the resistance to BET inhibitors [91]. Conversely, miR-150 or miR-26a expression/function restoration, BET inhibitors, and IRAK1/4i have been shown to downregulate MYC [73, 89], thereby suggesting a therapeutic potential for MLL-rearranged leukemias. Intriguingly, concomitant IRAK1/4 and BET inhibition has synergistic effects [88]. C-Myc, as a critical factor for DOT1L-associated transcriptional activation, is essential for the presence of DOT1L and H3K79me2 at several genomic loci. On the other hand, DOT1L, as one main component, may be involved in c-Myc-dependent transcriptional and epigenetic regulation [92, 93]. Therefore, DOT1L and c-Myc may play a synergistic role in promoting the activation of target genes. The dual targeting of DOT1L and c-Myc is also expected to improve therapeutic outcomes for MLL-rearranged leukemias [67], but this combination needs to be confirmed experimentally.

Furthermore, BCL-2 inhibitors, such as venetoclax (ABT-199), a highly specific inhibitor of chronic lymphocytic leukemia [94, 95], have shown synergistic effects against MLL-rearranged leukemias in combination with DOT1L inhibitors.

### DOT1L and immunotherapy

Epigenetic therapy combined with adoptive cellular therapy (ACT), including T cell receptor (TCR) or chimeric antigen receptor (CAR) T cell therapy, has become a highly used treatment for hematologic malignancies. Tumors may be exposed to epigenetic therapy prior to ACT, and/or T cells may be preconditioned with epigenetic therapy prior to reinfusion [96]. For example, enhancer of zeste 2 (EZH2), LSD1, or histone deacetylase (HDAC) inhibitors may be used to pretreat patients and create an inflammatory environment prior to reinfusion, thus improving CAR T cell manufacturing and antitumor efficacy [97, 98]. However, there are no reports about the utilization of DOT1L inhibition during the manufacturing of CAR T cell products to improve the antitumor efficacy.

DOT1L has been reported to be recruited to proinflammatory cytokine promoters, such as IL6 and IFN-β, in macrophages and then mediate H3K79me2/3 modifications at these sites to facilitate the transcription and expression of IL6 and IFN-β, which are essential to fully activate innate immune responses [99, 100]. Furthermore, DOT1L-mediated H3K79me2 has been shown to limit the Th1-cell gene program and IFN-γ expression in CD4 + T cells [101]. Instead, DOT1L inhibition may decrease the above reaction. Therefore, DOT1L and DOT1L-mediated H3K79 methylation may be involved in the inflammatory response and innate immunity. However, whether DOT1L-dependent immunoregulation plays an important role in tumor pathogenesis or antitumor immunity remains unclear. Remarkably, DOT1L inhibition has been shown to increase the dual-specificity phosphatase 6 (DUSP6) ERK phosphatase to attenuate xenogeneic graft versus host disease development and selectively ameliorate low-avidity T cell responses; moreover, DOT1L inhibition does not affect high-avidity antitumor T cell responses in xenogeneic and allogeneic adoptive immunotherapy models [102]. However, it remains to be demonstrated whether DOT1L inhibition promotes T cell stemness in ACT or improves CAR T cell manufacturing in vivo.

There is no comprehensive and systematic cross-comparison that is direct among the efficacies of the abovementioned combination therapies that are DOT1L epigenetic-based. Notably, many endeavors have been performed in this field. For example, the synergism of EPZ-5676 with the inhibition of DNMT, HDAC, histone demethylase, standard of care drugs, and bromodomain has been observed, and the results showed a synergy for BRD and LSD1 inhibitors depending on the specific inhibitor used, consistent synergy for DNMT inhibitors and standard of care drugs, and unsatisfactory results for HDAC inhibitors [83]. Synergism of EPZ004777 with menin inhibition was reported to be equally or more effective than a combination of DOT1L inhibitor with cytarabine, while EPZ004777 with BRD4 inhibition demonstrated moderate synergy. Therefore, a menin inhibitor is more suitable as a partner of DOT1L than as an individual to treat MLL-rearranged leukemias [67]. However, most of the above studies mainly focus on MLL-AF9 or AF4 leukemic cells; for many other MLL subtypes, it is unclear whether these treatment regimens are appropriate. Encouragingly, two of the abovementioned combinational therapeutic regimens have entered clinical trials (NCT03701295 and NCT03724084) (Table 1). Furthermore, it may be interesting and meaningful to explore the role of targeting DOT1L in CAR T cell therapy, especially for relapsed and refractory cases, e.g., B-lymphoblastic leukemia and MLL rearranged leukemia.

## Problems, challenges, and outlook

### DOT1L inhibitors need to be further developed

Although medicinal chemistry fields have experienced great progress to date, such as the development of EPZ-5676, developing DOT1L inhibitors with high potency, selectivity, and good pharmacokinetics still needs much work. Fortunately, the existing findings provide valuable insights [22].

### Small molecule DOT1L degraders are worth trying

As an emerging therapeutic strategy, DOT1L degraders such as DOT1L-targeting PROTACs are worth exploring as an alternative, but many challenges exist. In recent years, progress has been made in the development of small molecule degraders for hematologic malignancies. For example, VHL and CRBN, which have been developed as E3 ligases to design PROTACs or molecular glues against BCL-XL; Bruton’s tyrosine kinase, IKZF1, and GSPT1 for hematologic malignancies, including B cell malignancies; and MM, AML, and CML (e.g., NCT04886622, NCT04830137, NCT04756726, NCT04434196, NCT02848001). These clinical studies will provide proof-of-principle that small molecule degraders can be developed as effective therapeutics for hematologic malignancy, and these advances will also provide valuable insights for developing small molecule DOT1L degraders.

### Drugs targeting PPIs that involve DOT1L are under exploration

Recently, many small molecule PPI inhibitors, such as MLL1-menin and MLL1-WDR5 interaction inhibitors, were synthesized and entered into clinical trials for AML, suggesting that PPIs involving DOT1L may have similar potential for MLL-rearranged leukemias. The current understanding and knowledge of the PPIs that involve DOT1L and other chromatin modifiers, including their structure, structure–function relationships, and the mechanisms of activating gene transcription and leukemia transformation, provides a rationale for developing these PPI inhibitors. However, the pharmacochemistry of these PPIs is relatively backward, and drug-like small molecule inhibitors are desirable and worth exploring [45].

There are some additional problems that deserve attention. First, because DOT1L may interact with many different proteins, there are different PPI inhibitors. Thus, the efficacy of these inhibitors needs to be compared to select better inhibitors, but there are no similar comparative studies. Second, PPI inhibitors are designed to target the binding sites of DOT1L itself or DOT1L-interacting proteins, but which of these inhibitors exerts better activity is not known. Third, it remains to be determined whether such PPI inhibitors are more effective than DOT1L enzymatic inhibitors. Fourth, these PPI inhibitors may also affect similar interactions among chromatin proteins other than DOT1L, resulting in off-target or side effects. Overall, at present, there is a lack of systematic preclinical studies on these PPI inhibitors in the treatment of MLL-rearranged leukemias. Therefore, further biological activity and medicinal chemistry investigations of such PPI inhibitors are warranted.

### DOT1L epigenetic-based combination therapy has promising prospects

In this review, we summarized the recent results of DOT1L suppression combined with other regimens, including conventional chemotherapy, targeted therapy, immunotherapy, and other therapeutic schemes, and we expounded the underlying rationale of these combinational therapies. However, several interesting questions remain. Similar to permutations and combinations in mathematics, there are many options for adopting combination therapies that contain DOT1L, but the best form is unknown. Notably, EPZ-5676 combined with standard of care drugs and hypomethylating agents, which was reported to be similar to DOT1L-menin inhibition [67], has entered clinical trials, and efficacy has not been reported yet. However, methods targeting DOT1L together with menin inhibition and other sensitizers, such as AF9 and SIRT1 activator, have not yet entered clinical trials. Furthermore, it is unknown whether DOT1L inhibition occurs by enzymatic inhibition or PPI inhibition in a specific combinational therapy. A similar concern may be encountered with other therapeutic regimes. Therefore, it is important to select the appropriate combinational therapeutic regimens to ensure synergistic cooperation. For example, combining DOT1L inhibitors with SIRT1 activators rather than SIRT1 inhibitors improves the antitumor activity; otherwise, the clinical benefit would be reduced due to the antagonism of their downstream effect [71].

There are many combination therapies for MLL-rearranged leukemias that do not contain the DOT1L1 target, and the possibility that some of these therapies are probably more effective cannot be excluded. It is unknown which patients are suitable for regimens that involve the DOT1L1 target. Comparing the efficacy of these different combinational schemes would be helpful to guide the treatment of MLL-rearranged leukemias. It is well known that combinational therapy should be based on a comprehensive molecular rationale, especially when manipulating epigenetic regulators in a context-dependent manner; an example is the considerable insight gained into the dynamic interplay between chromatin regulators or the epigenetic networks controlling the activation and repression of gene expression [96, 103]. Therefore, understanding the patient's MLL gene rearrangement and fusion genotype as well as the possible molecular mechanism of DOT1L and other composite targets will help to determine the effective dual-targeting options for an individual and precise treatments.

## Conclusions

We presented a general overview of therapeutic strategies against DOT1L for MLL-rearranged leukemias, including DOT1L enzymatic inhibitors, DOT1L degraders, PPI inhibitors, and combinatorial interventions. In addition, the existing concerns, challenges and prospects of the different therapeutic approaches were discussed. Although a variety of DOT1L enzymatic inhibitors have been identified, most of them require further optimization. Recent advances in small molecule degraders provide valuable insights for designing DOT1L degraders, but support through the development of medicinal chemistry is needed. To block DOT1L-associated PPIs, drug R&D strategies and platforms need to be developed, and preclinical experiments need to be performed. DOT1L epigenetic-based combination therapy has attracted increasing attention, and a thorough understanding of the regulatory mechanism of DOT1L epigenetic modification will contribute to the formulation of clinical treatment schemes. We hope that this study will provide valuable guidance for DOT1L-associated drug development and future clinical treatments of MLL-rearranged leukemias.

## Data Availability

Not applicable.

## Abbreviations

MLL: Mixed lineage leukemia
AML: Acute myeloid leukemia
ALL: Acute lymphocytic leukemia
DOT1L: Disruptor of telomeric silencing 1-like
H3K79: Histone H3 lysine 79
H3K79me1: Histone 3 lysine 79 monomethylation
H3K79me2: Histone 3 lysine 79 dimethylation
H3K79me3: Histone 3 lysine 79 trimethylation
ENL: Eleven-nineteen lysine-rich leukemia
CC: Coiled-coil
DotCom: DOT1L-containing complex
RNA Pol II: RNA polymerase II
EAPs: ENL-associated proteins
P-TEFb: Positive transcription elongation factor b
AEP: AF4 family/ENL family/P-TEFb
SEC: The super elongation complex
SAM: (S)-adenosyl-l-methionine
SAH: (S)-adenosyl-l-homocysteine
BET: Bromodomain and extraterminal domain
DHODH: Dihydroorotate dehydrogenase
VS: Virtual screening
FBDD: Fragment-based drug discovery
HTS: High-throughput screening
TPD: Targeted protein degradation
PROTACs: Proteolysis-targeting chimeras
POI: Protein of interest
PPIs: Protein–protein interactions
H3K9ac: Histone H3 acetyl Lys9
YEATS: Yaf9, ENL, AF9, Taf14, and Sas5
NMR: Nuclear magnetic resonance
OM-LZ: Octapeptide motif-leucine-zipper
CALM: Clathrin assembly lymphoid myeloid leukemia
GAS41: Glioma-amplified sequence 41
H2BK120ub: Histone H2B lysine 120 monoubiquitination
RNF20: Histone H2B E3 ubiquitin ligase ring finger protein 20
H4K16ac: Acetylation of histone 4 lysine 16
CTD: C-terminal domain
NPM1: Nucleophosmin
SIRT1: Silencing information regulator 2 related enzyme 1
SUV39H1: Suppressor of variegation 3–9 homolog 1
BRD4: Bromodomain containing 4
EP300: E1A binding protein
LSD1: Lysine-specific histone demethylase 1
DNMT: DNA methyltransferase
MDS: Myelodysplastic syndrome
CDK9: Cyclin-dependent kinase 9
AFF1: AF4/FMR2 family member 1
AFF4: AF4/FMR2 family member 4
ACT: Adoptive cellular therapy
TCR: T cell receptor
CAR: Chimeric antigen receptor
EZH2: Enhancer of zeste 2
HDAC: Histone deacetylase
DUSP6: Dual-specificity phosphatase 6
